# Host–Microbiome Interactions as Moderators of Host Quality and Biodiversity–Disease Relationships

**DOI:** 10.1093/icb/icaf091

**Published:** 2025-06-12

**Authors:** Kristen M Rosamond, Helen Joan Esser, Rebekka B Assink, Laura Jaramillo-Ortiz, Melissah Rowe, Ellie C Kirke, Kevin D Matson

**Affiliations:** Department of Biology, University of Missouri-St. Louis, St. Louis, MO 63121, USA; Wildlife Ecology and Conservation Group, Wageningen University & Research, 6708 PB Wageningen, The Netherlands; Wildlife Ecology and Conservation Group, Wageningen University & Research, 6708 PB Wageningen, The Netherlands; Department of Wildlife and Environment, Van Hall Larenstein University of Applied Sciences, 8934 CJ Leeuwarden, The Netherlands; Wildlife Ecology and Conservation Group, Wageningen University & Research, 6708 PB Wageningen, The Netherlands; Centre for Infectious Disease Control, National Institute for Public Health and the Environment, 3720 BA Bilthoven, The Netherlands; Department of Animal Ecology, Netherlands Institute of Ecology (NIOO‐KNAW), 6700 AB Wageningen, The Netherlands; Wildlife Ecology and Conservation Group, Wageningen University & Research, 6708 PB Wageningen, The Netherlands; Development Economics Group, Wageningen University & Research, 6708 PB Wageningen, The Netherlands; Wildlife Ecology and Conservation Group, Wageningen University & Research, 6708 PB Wageningen, The Netherlands

## Abstract

Biodiversity–disease studies typically focus on how changes in community composition (e.g., species richness, abundance, and functional or phylogenetic metrics of biodiversity) affect disease risk. In doing so, these studies tend to overlook intraspecific variation in the organisms comprising the community. Yet, intraspecific variation, which occurs to varying degrees both within and between communities, could also modulate biodiversity–disease relationships. One important driver of intraspecific variation is the microbiome. By directly and indirectly influencing health and susceptibility to infection and disease, microbiomes are integral to organismal functioning. Thus, the microbiome plays a crucial role in host quality. We define host quality as an integration of host traits related to parasite exposure, establishment, growth, and infectivity, which ultimately shape parasite fitness. The microbiome can impact host quality via a variety of mechanisms including host size and developmental stage, immune function, reproduction, nutrient acquisition, and behavior. However, the potential for such microbiome-driven changes in host quality to trigger cascading effects on community-level processes, specifically by altering parasite transmission dynamics and community competence, has not been well explored. Here, we examine and illustrate a pathway by which the microbiome may influence variation in organismal biology (i.e., host quality) of hosts in communities. Furthermore, we consider how major anthropogenic drivers of microbiome shifts, such as climate change, pollution, land use change, and domestication, might influence this pathway and thereby alter outcomes. Future studies bridging microbiome and disease ecology research will provide opportunities to unify these concepts across scales and between the plant and animal domains. To date, most microbiome research has focused on humans, crops, and laboratory animals. However, to better understand the potential for knock-on ecological effects of microbiomes, more attention must be paid to the microbiomes of wild plants and animals. Ultimately, more experimental and theoretical data are needed to clarify how the microbiome impacts host quality and disease dynamics, as well as how anthropogenic factors continuously reshape these relationships.

## Introduction

Biodiversity–disease studies typically explore how changes in **biodiversity impact** disease risk in ecological communities (Keesing et al. 2006; Johnson et al. 2013; Mahon et al. 2024; bolded terms defined in Table 1, Glossary). Because these studies generally assume that species-level differences (interspecific variation) are the primary driver of disease risk, they tend to overlook differences among individuals within species (intraspecific variation). Yet, intraspecific variation—both within and between communities—can also shape disease risk. One key source of intraspecific variation is the **microbiome** (i.e., **microbiota** plus genetic material). However, while microbiomes are increasingly recognized as fundamental drivers of biological variation among hosts, including **infection** and disease, they are rarely incorporated into the biodiversity–disease framework.

**Table 1:** Glossary.

Term	Definition
Biodiversity/diversity	The variety of life found in a specific place; can be characterized at different spatial scales (from local to global) and levels of biological organization (from genes to ecosystems) using multiple, sometimes inter-related metrics (e.g., species richness, abundance, and functional or phylogenetic metrics of biodiversity). For clarity, we use the term “biodiversity” to refer to the variety of life found at the host level and the term “diversity” to refer to the variety of life at the microbiome level.
Biodiversity–disease relationship	How biodiversity impacts parasite transmission and disease risk.
Community	A group of interacting populations of different species occurring together in space; sometimes used in disease ecology as a shorthand for assemblage, i.e., a *taxonomically related* group of interacting populations of different species occurring together in space (Stroud et al 2015). We use the term “community” to refer to communities of host species rather than communities of microbes found within hosts.
Community competence	A community's capacity to support infection (Johnson et al 2013); here, refers to a community of host species (see “community”).
Community composition	All (potential) host- and non-host-species (i.e., encompassing the full range of host quality) in a community, often quantified simplistically using the metrics of biodiversity; here, refers to a community of host species (see “community”).
Disease	Any harmful deviation from the normal structural or functional state of an organism, associated with specific signs and symptoms; sometimes used synonymously with infection (see “biodiversity–disease relationship”).
Dysbiosis	Any change to the composition of resident commensal microbes relative to the microbiome found in healthy individuals (Petersen and Round 2014).
Establishment	A component of host quality representing the probability that a parasite successfully infects a host, given contact has occurred.
Exposure	A component of host quality representing the probability of contact between a host and infectious parasite propagules.
Growth	A component of host quality representing the growth and persistence of a parasite in an infected host.
Host quality	A host species’ contribution to the fitness of a parasite species. We employ the model of host quality proposed by Mommer et al. that defines host quality via the components of exposure, establishment, growth, and infectivity. Host quality can be viewed as a spectrum, with a “non-host” having a host quality of zero (i.e., no contribution to parasite fitness), a low-quality host contributing relatively little to (or even reducing) parasite fitness, and a high-quality host enhancing parasite fitness.
Infection	An invasion of the body tissues of a host by an exogenous agent, regardless of whether or not it causes disease.
Infectivity	A component of host quality representing infectious propagules released from a host, which are then accessible to susceptible hosts.
Microbiome	The collection of genomes from all microorganisms found in a given environment, i.e., the microbiota plus the genetic material associated with it, including products of genes. Following Rowe et al. 2020, we use this more inclusive term throughout this paper rather than “microbiota.”
Microbiota	The community of living microorganisms found in a given environment.
Parasite	A microorganism that lives in or on a host at the expense of the host; we use the term “parasite” as a blanket term for infectious disease-causing agents.
SIR models	Mathematical models of infectious disease incorporating compartments of susceptible (S), infectious (I), and recovered (R) individuals.

Microbiomes are associated with myriad facets of host biology including immunity, development, and energy regulation and metabolism. Most microbiome studies have focused on humans, model organisms, crops, and domesticated animals, while wild animal and plant microbiomes remain vastly understudied (Carding et al. 2015; Hanning and Diaz-Sanchez 2015; Coleman-Derr et al. 2016; Carrión et al. 2019). While an increasing number of studies in plants and animals have highlighted the microbiome's role in disease (Bozzi et al. 2021; Gu et al. 2022), such research often remains disconnected from broader disease ecology (but see plant-soil feedback framework, e.g., Collins et al. 2020). Most microbiome studies focus on individuals within a single host species, while disease ecology has historically examined **parasite** transmission at broader scales, such as populations (Anderson and May 1981; Meyer et al. 2022) and communities (Ampt et al. 2022; Stewart Merrill et al. 2022). Disease ecology studies often focus on how the immune system interacts with parasites within hosts (Pedersen and Fenton 2007) as well as population-level effects of infection (Childs et al. 2019) but do not directly connect these interactions to processes at the **community** and assemblage level. This omission may inadvertently mask mechanisms by which variation in traits like microbiome composition influence parasite transmission.

**Fig. 1:**
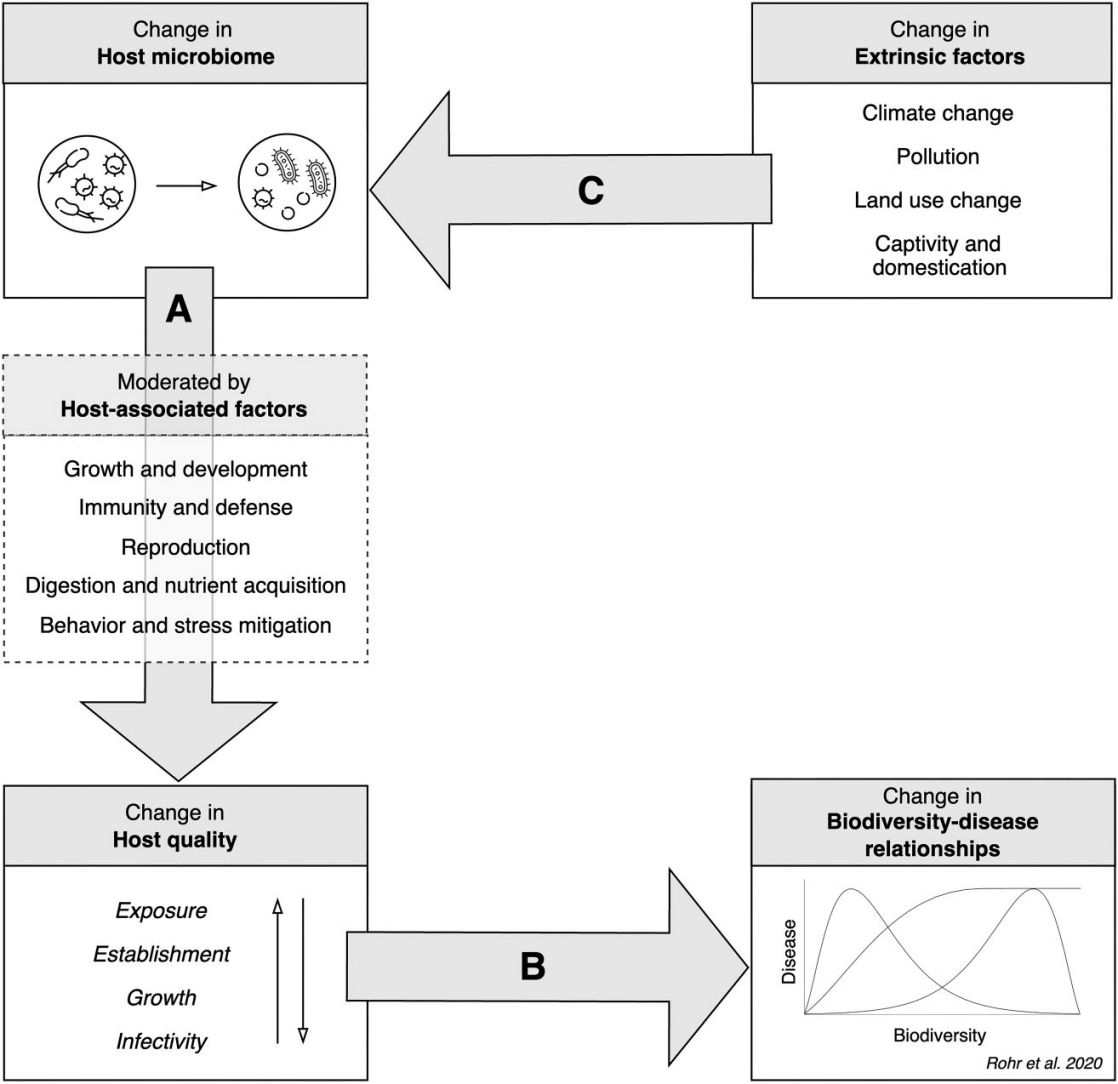
Overview of the pathway proposed in this paper. Changes in the host microbiome can drive changes in host quality (arrow A), which in turn can impact biodiversity–disease relationships (arrow B); this pathway can be influenced by host-extrinsic (here, anthropogenic) factors (arrow C). (A) Changes to host microbiome composition can either increase or decrease any of the four components of host quality (parasite exposure, establishment, growth, and infectivity). These increases or decreases can be moderated, either directly or indirectly, by various host-associated factors (dashed box). (B) Changes in host quality can subsequently affect biodiversity–disease relationships in numerous hypothetical ways. A figure from Rohr et al. (2020) illustrates potential relationships between biodiversity and disease. (C) Extrinsic factors can influence host microbiomes, triggering cascading effects that ultimately influence biodiversity–disease relationships (i.e., via A and B). The structure and sequence of this pathway, depicted through boxes and arrows, are mirrored in the main sections of the accompanying text.

Here, we explore how host–microbiome interactions may trigger cascading effects that impact community-level processes, specifically by influencing **biodiversity–disease relationships**. First, we examine how microbiomes impact infection, disease, and **host quality** (Fig. 1A), thereby affecting parasite transmission. While interactions between the microbiome and host-associated factors (e.g., growth, immunity) are bidirectional, we focus on how the microbiome shapes such characteristics to better understand its influence on host quality. We examine the impacts of the microbiome on host quality using a new model (Mommer et al., in review) that focuses on four components shaping parasite fitness: parasite **exposure, establishment, growth**, and **infectivity** (components of the Mommer model are italicized hereafter). We note that these terms are used to describe parasite, not host, fitness. Specifically, “*establishment*” refers to establishment of a parasite on a host (rather than establishment of a host in a habitat), and “*growth*” refers to parasite growth (rather than host growth). Second, we explore scenarios in which microbiome-mediated shifts in host quality could impact biodiversity–disease relationships at the community level (Fig. 1B). Third, we examine how host-extrinsic factors, with an emphasis on anthropogenic disturbances, can alter host microbiomes and thereby impact parasite transmission via changes in host quality (Fig. 1C). Finally, we reflect on the types of studies needed to expand knowledge of the role of the microbiome in biodiversity–disease relationships and thus support or refute the conceptual framework that we present here. In building this framework, we primarily focus on wild species of plants and animals but also incorporate ideas from the more extensive microbiome literature in humans, domesticated animals, and crops.

## Microbiomes influence infection, disease, and host quality

### Microbiomes influence infection and disease

Links between microbiome variation and infection and disease have been identified in humans, but, given their complexity, many causal relationships have yet to be shown (Carding et al. 2015). In wild plants and animals, where research is typically limited to correlative studies, this relationship is even more poorly understood. Having a diverse microbiome is sometimes associated with better health, a pattern observed across humans (Manor et al. 2020), animals (e.g., McKenney et al. 2017), and plants (Saleem et al. 2019). However, focusing solely on microbiome **diversity** (see Table 1, Glossary) is overly simplistic, as the relationship between good health and microbiome diversity can vary (Huttenhower et al. 2012). Also, diversity can be measured at various scales (e.g., alpha, beta, or gamma diversity) and in different ways (e.g., taxonomic or functional diversity), adding to the challenge of understanding the relationship between microbiome diversity and health. Beyond diversity, specific microbes can often confer distinct health benefits. For example, keystone taxa, which are rare species with disproportionate effects on the microbiome, are essential for maintaining organismal health and function (Banerjee et al. 2018).

While the microbiome can promote host health, it can also facilitate the invasion of parasites; in addition, changes in resident microbes, such as overgrowth, can lead to infection (Stevens et al. 2021). Existing research shows that changes in the microbiome sometimes precede disease, suggesting that the microbiome could be used as an indicator of disease in some systems (Gu et al. 2022; Ribas et al. 2023). Such changes to the microbiome can include loss of diversity, overgrowth of harmful microbes, and reduction in beneficial microbes, which can result in **dysbiosis** and increased disease susceptibility (Carding et al. 2015; Levy et al. 2017; Stevens et al. 2021). While the complex, bidirectional nature of microbiome–disease relationships makes it difficult to determine causation, the microbiome clearly plays a role in organismal health and disease. Thus, in the same spirit as studies suggesting the inclusion of the microbiome in classic disease models (e.g., the disease triangle) to better understand parasite transmission (Bernardo-Cravo et al. 2020), we argue for incorporating the microbiome into biodiversity–disease relationships.

### Microbiomes influence host quality

#### Growth and development

The microbiome plays a critical role in growth and development across diverse host taxa, which can translate into variation in host quality. One key pathway is through body size, which can vary among conspecifics of the same age due to microbiome-driven developmental differences. Host body size is positively correlated with both parasite species richness (Kamiya et al. 2013) and parasite biomass (Poulin and George-Nascimento 2007) across a wide range of taxa. Larger hosts may encounter parasites more frequently and have more surface area for colonization, which are two mechanisms enabling greater parasite *exposure*.

Early life conditions, both microbiome-dependent and independent, can shape host physiology in ways relevant to disease processes. For example, in the yellow fever mosquito (*Aedes aegypti*), gut microbes influence the speed of larval development (Roman et al. 2024), and a slower larval growth rate favors horizontal transmission of parasites over vertical transmission (Agnew and Koella 1999), potentially impacting *infectivity*. Experimental approaches that remove or add microbes illustrate the microbiome's role in development particularly well. For instance, introducing a *Wolbachia* bacterium strain into yellow fever mosquitoes accelerated larval development, reduced lifespan, and altered body size while concurrently interfering with viral transmission (Yeap et al. 2011). In addition, in a grass species (*Setaria viridis*), experimental reduction of the seed microbiome reduced seed vigor, an integrative measure of fitness that incorporates aspects of development (Rodríguez et al. 2020). Furthermore, in a test of critical developmental windows in wood frogs (*Rana sylvatica*), experimental sterilization of eggs and subsequent inoculation with gut microbes from another frog species increased both growth and survival of larvae exposed to ranavirus compared to sterilized, un-inoculated controls (Warne et al. 2019). Individuals with compromised early development experienced increased mortality, suggesting altered host quality, though the precise mechanism (and thus related component(s) of host quality) remains unclear.

Finally, an example involving plants demonstrates the microbiome's potential effects on each component of host quality via changes in growth and development. In ribwort plantain (*Plantago lanceolata*), experimental inoculation with mycorrhizal fungi resulted in increases in both host growth rate and the rate of infection by a fungal parasite (Eck et al. 2022). Thus, in this system, larger plants might encounter and disperse more wind-dispersed parasitic spores (*exposure* and *infectivity*), provide more microhabitats suitable for spore germination (*establishment*), and allocate less energy to control parasite *growth* in favor of investing in biomass.

#### Immunity and defense

Research has shown that the microbiome influences the immune system across diverse taxa, which can directly impact host quality. Studies have associated animal and plant microbiome composition with infection status (Bozzi et al. 2021), disease severity and resistance (Pieterse et al. 2014; Namasivayam et al. 2019), and various immunological measures (e.g., Leclaire et al. 2014; Chen et al. 2023). Mechanisms of immunity in animals and plants are complex, vary across taxa, and are detailed elsewhere (see Jones and Dangl 2006; Morella and Koskella 2017). Here, we instead focus on how the microbiome may influence host quality via immunity.

Host-associated microbiomes can decrease host quality by interfering with both parasite *establishment* and *growth* via a mechanism called colonization resistance (van der Waaij et al. 1971), which appears to be present across various taxa (Chiu et al. 2017). Higher colonization resistance against parasites has been associated positively with microbiome diversity (Rybakova et al. 2017) and negatively with compromised immunity (Sprinz et al. 1961; Willing et al. 2011). In cases where a parasite successfully establishes, the microbiome can still help suppress its *growth*. For example, if fungal root parasites breach a plant's first line of defense in the rhizosphere, endosphere microbes can become enriched, helping to limit further parasite *growth* (Carrión et al. 2019). Resident microbes can also restrict parasite *growth* by helping to contain parasites within a given location inside of the host and prevent spread (Chiu et al. 2017), a mechanism that has been illustrated in studies of laboratory mice (Martins et al. 2013) and appears to occur in mucosal surfaces across diverse taxa (Barr et al. 2013). In some cases, functional roles for certain microbes have also been established, for example in microbes isolated from bat skin that inhibit *growth* of *Pseudogymnoascus destructans*, the fungus causing white-nose syndrome (Hoyt et al. 2015).

Microbiomes can also facilitate infection and, in doing so, increase host quality (Stevens et al. 2021). For example, increasing the prevalence of a core resident microbe in the honey bee (*Apis mellifera*) gut makes bees more susceptible to parasitic infection (Schwarz et al. 2016). In addition, it is possible for viruses to exploit gut microbes to increase their own replication and transmission, which has been demonstrated in laboratory mice (Kuss et al. 2011). These two examples highlight how microbes can increase host quality (i.e., increase parasite fitness), in contrast to the prior examples in this section showing how microbes can decrease host quality (i.e., decrease parasite fitness).

#### Reproduction

Microbes’ influence on host reproduction has long been recognized within the framework of parasite-mediated sexual selection and female mate choice (Hamilton and Zuk 1982; Borgia and Collis 1990), including models suggesting that females use phenotypic traits to avoid mating with infected males (Able 1996; Loehle 1997). More recently, animal microbiomes have been shown to impact reproductive function and processes in diverse ways (Rowe et al. 2020; Comizzoli et al. 2021), some of which may generate variation in host quality. Notably, microbiomes may impact the probability of contact between infected and uninfected hosts, and thus host *exposure*, by influencing mating behavior. Microbes can alter host mating patterns in various ways, including via effects on mate attractiveness, mating preferences, and mating performance (e.g., Dass et al. 2011; Heys et al. 2018; Yi and Cha 2022). Such effects can be driven by microbiome impacts on host physiology. For example, the gut microbiome can influence the regulation and degradation of reproductive hormones (Hussain et al. 2021). In turn, hormone levels can shape factors like copulation (Wingfield 1984) and plumage coloration (Khalil et al. 2020) that influence mating activity and mate choice. Similarly, microbiome-mediated olfactory cues may facilitate mate choice by signaling information about a host's sex, breeding status, or social status (Theis et al. 2013; Brunetti et al. 2019). Moreover, in many invertebrates, symbiotic microbes can distort sex ratios (Engelstädter and Hurst 2009), with potential impacts for host mating patterns (e.g., female promiscuity, Charlat et al. 2007).

Importantly, the impact of microbiomes on mating decisions can reduce host *exposure*, for example, when microbiome-mediated cues allow individuals to minimize mating encounters with infected individuals. Conversely, when microbiomes increase overall mating rates or increase attractiveness of infected individuals, host *exposure* is likely to increase. Intriguingly, increased mate attractiveness may reflect a strategy of the microbiome to increase microbe transmission and infection rates (Rowe et al. 2020). Finally, reproductive microbiomes may directly influence host quality. For example, the human vaginal microbiome can both prevent *establishment* of opportunistic infections and limit their *growth* (France et al. 2022). However, whether similar effects occur in other animals, and particularly in wild species, remains unknown.

Plant-associated microbiomes may also influence host quality through effects on reproduction. For example, the soil microbiome can impact flowering time (Wagner et al. 2014; Chaney and Baucom 2020), potentially affecting pollinator-transmitted disease dynamics. Importantly, microbiome-mediated variation in flowering times could influence host *exposure*. In the white campion (*Silene latifolia*), earlier flowering individuals are more likely to be infected by the pollinator-transmitted anther-smut fungus (*Ustilago violacea*) (Biere and Antonovics 1996). While the microbiome's role in the white campion's flowering time is unclear, microbiome-driven variation in reproductive traits and its impact on plant host quality warrants further investigation.

#### Digestion and nutrient acquisition

Microbes in animals, particularly in the gut, play essential roles in digestion and energy metabolism (Hanning and Diaz-Sanchez 2015). By facilitating digestion and nutrient acquisition, the microbiome provides hosts with energy to function at optimal capacity. This could either decrease host quality (e.g., by boosting immune defense and helping hosts resist infection) or increase host quality (e.g., by providing parasites with more nutrients to support their own *growth* and reproduction) (Civitello et al. 2018). Though causation is often unclear, diet quality has been correlated with changes in the animal microbiome in numerous cases (e.g., Kohl et al. 2016; Sugden et al. 2020), and it can influence an animal's resistance or tolerance to parasites (Ezenwa 2004; Dolezal et al. 2019). The microbiome also enables the digestion of otherwise indigestible compounds (e.g., Ceja-Navarro et al. 2019), as well as parasitic microbes that could directly cause disease. For instance, vultures host antilisterial bacteria that may help prevent parasite *establishment* and *growth*, allowing them to safely consume carrion containing parasitic microbes (Zepeda Mendoza et al. 2018).

In plants, the microbiome enhances nutrient uptake, such as by aiding in nitrogen fixation (Trivedi et al. 2020). While the relationship between nutrition and disease in plants is complex, it is clear that nutrition—potentially mediated by the microbiome—affects disease (Chapin 1980; Walters and Bingham 2007). Research links microbiome composition to imbalances in essential minerals like calcium and magnesium, which likely increases abiotic stress (Lazcano et al. 2021), as an appropriate balance of calcium and magnesium is crucial for plant immunity (Huber and Jones 2013). Therefore, it appears that microbiome changes can influence disease in plants by altering nutrient availability and ultimately affecting parasite *establishment* and *growth*, which is potentially mediated by other host-associated factors including growth and immunity.

#### Behavior and abiotic stress mitigation

Social behavior affects the spread of microbes that impact host health and disease, including viruses, antibiotic-resistant microbes, and microbes involved in metabolism and immunity (Sarkar et al. 2024). The microbiome has been linked to variation in behavior-related traits, which can influence host quality, primarily via *exposure*. For instance, altered social interactions could impact the type and quantity of encounters between individuals, and thus *exposure* to parasites (as discussed in the "Reproduction" section). For example, microbiomes can mediate olfactory cues via pheromones that influence aggregation behavior (Wada-Katsumata et al. 2015). The microbiome has also been linked to dispersal (Goodacre et al. 2009) and exploration (Florkowski and Yorzinski 2023), which may then influence host quality by impacting *exposure* to parasites. For example, a recent study in white-footed mice (*Peromyscus leucopus*) found that individuals moving farther distances exhibited higher tick parasitism, implicating increased *exposure* as a potential mechanism (Brehm et al. 2025). In addition, inoculating germ-free mice with microbes from various wild rodent species altered their diet preferences (Trevelline and Kohl 2022), indicating that the microbiome impacts diet selection and therefore foraging behaviors in the wild. This suggests a potential mechanism by which the microbiome can influence host quality, as different foraging behaviors can result in different *exposure* risks.

While plants do not exhibit behaviors in the same way as animals, the plant microbiome has an important role in facilitating responses to abiotic stress such as drought, salinity, and nutrient limitation (Caddell et al. 2019). Such abiotic stresses can predispose plants to disease (Bostock et al. 2014), but the microbiome can potentially decrease host quality via abiotic stress mitigation. For example, van der Voort et al. (2016) demonstrated that heat disturbance of soil led to a reorganization of rhizobacterial communities, which was associated with changes in disease suppression. This established a clear connection between the microbiome and host quality that may be moderated by changes in abiotic stress mitigation.

## Host–microbiome interactions may trigger cascading effects on biodiversity–disease relationships

Early studies of biodiversity–disease relationships focused on agriculture, recognizing that simplified (monoculture) systems are more susceptible to disease outbreaks than complex (natural) systems (Elton 1958). Over time, biodiversity–disease research has expanded to include wild animal and plant communities. Despite this expansion, biodiversity–disease research still primarily focuses on relationships centered on host **community composition** (see Table 1, Glossary). Here, after briefly describing how changes in community composition can shape biodiversity–disease relationships, we propose an additional pathway for changes in **community competence** based on changes in the microbiome.

### Changes in community composition can alter community competence

**Fig. 2:**
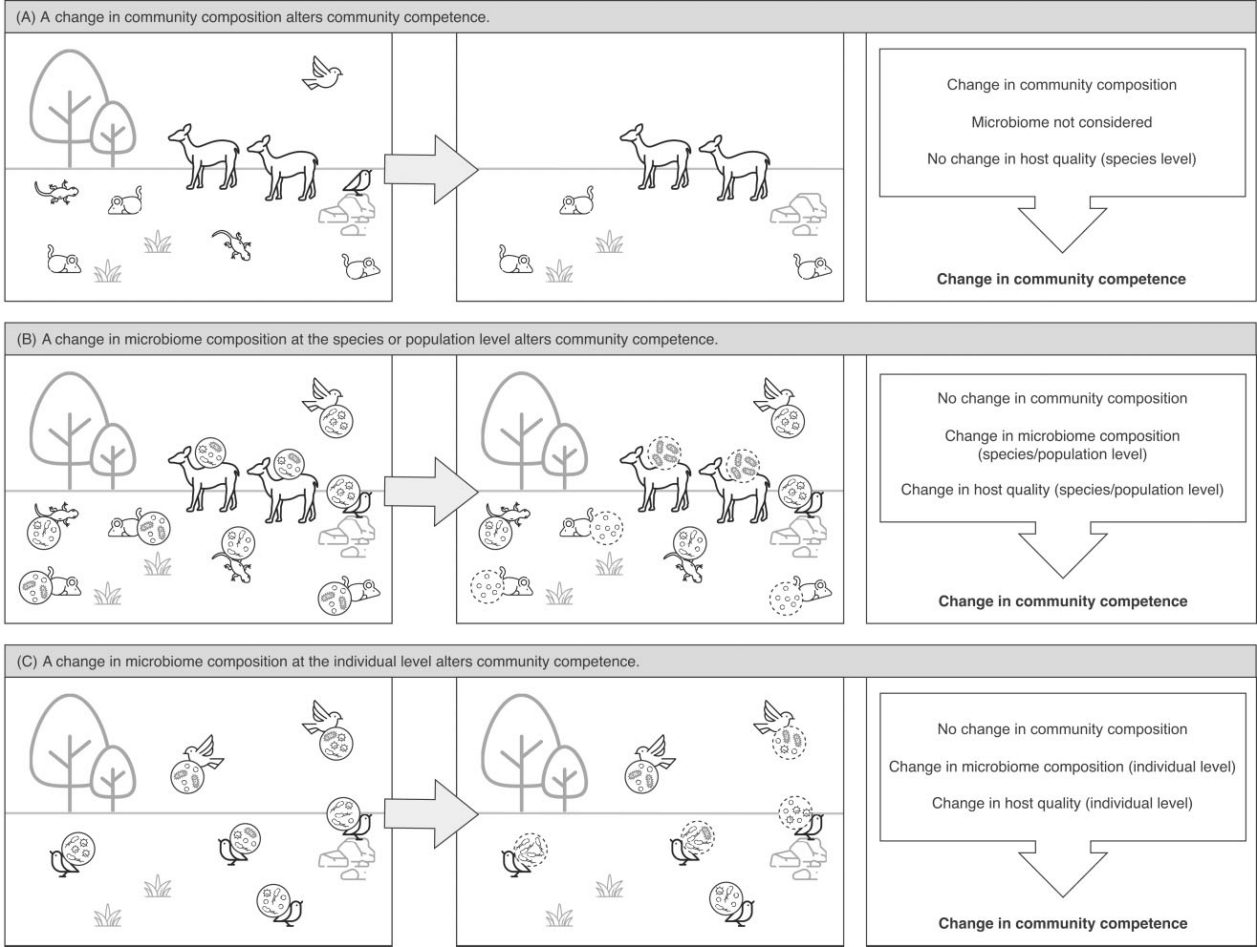
A shift in community competence may arise via various mechanisms, including changes in community composition or changes in microbiome composition. (A) This simple hypothetical community includes four vertebrate host species. One or more drivers (here, land use change, depicted as the loss of trees) change the ecosystem structure, resulting in the loss of two host species (lizard and bird). This shift in community composition can alter community competence without changes in the remaining species’ (rodent and deer) host quality. This scenario illustrates the conventional view of biodiversity–disease relationships in which changes in community composition can change community competence. However, via the pathway proposed in this paper, changes in community competence can also arise via changes in microbiome composition (B and C). (B) The initial community (from A) is depicted with host microbiome composition taken into consideration. Instead of a change in community composition, the community experiences a change in the microbiome composition (represented by dashed circles) of two host species (rodent and deer). This change in microbiome composition alters the host quality of the rodent and deer, resulting in a shift in community competence. In this simplified scenario, the microbiome is treated as a species- or population-level trait. (C) Focusing on a single member of a larger ecological community (i.e., a bird) is useful for capturing variation in microbiome composition among individuals within a species or population, offering a more accurate representation of community-level dynamics. Here, some individuals undergo a change in microbiome composition (represented by dashed circles), which can lead to either increased or decreased host quality in the affected individuals and thus altered community competence.

Biodiversity–disease studies typically focus on how changes in community composition via changes in species richness or abundance can alter community competence, or a community's capacity to support infection (Johnson et al. 2013). Foundational studies have illustrated how such changes (i.e., adding or removing host species from a community) can either dilute (reduce) or amplify (increase) disease risk (Keesing et al. 2006; Collins et al. 2020). We illustrate one example by which changes in community composition could alter community competence (Fig. 2A). In this scenario, land use change (i.e., trees removed from an ecosystem) leads to the loss of a lizard and bird species. This situation could hypothetically arise if the lizards depended on the removed trees for food, and the birds, in turn, depended on the lizards as a food source. Thus, the loss of the trees could result in the loss of both the lizard and bird species.

In this type of scenario, the species lost could either be high-quality hosts, which enhance parasite fitness, or low-quality hosts, which contribute relatively little to, or even reduce, parasite fitness. Whether the species lost are of high- or low quality determines the direction of the change in community competence. Loss of a high-quality host can reduce overall community competence, while loss of a low-quality host can increase community competence. Conversely, gaining a high-quality host can increase community competence, and gaining a low-quality host can decrease community competence. Other changes in community composition, such as in host species abundance, can similarly lead to an increase or decrease in community competence depending on the quality of the hosts in consideration. However, we argue that changes in community competence can also arise via other mechanisms not typically considered, namely changes in the host microbiome.

### Changes in microbiome composition at the species or population level can alter community competence

We propose that the microbiome has the potential to serve as a key trait influencing parasite transmission, much like (and perhaps interacting with) the life history traits of species (Sears et al. 2015) and contact rates within communities (Lloyd-Smith et al. 2005). Thus, similar shifts in how community composition influences community competence might be achieved through changes in the microbiome composition of a community's members, whether at the species or population level. In such a framework, the microbiome could be viewed as a species-level trait because it is shaped at least in part by other traits, such as diet type or sociality, that are typically characteristic of particular species. However, a similar pattern can also emerge at the population level, since populations may be exposed to different environmental factors (e.g., in rural versus urban habitats). In our depicted example (Fig. 2B), a change in the microbiome composition of populations in a community (illustrated by a change in rodent and deer microbiome composition) changes these populations’ host quality and results in a shift in community competence. These changes could come about by various hypothetical mechanisms. For instance, if land use change leads to more spatial overlap and interactions (even indirectly, for example, via food resources) between rodents and deer, the two species could start to share features of their microbiomes. If these species’ more homogenous microbiomes then increase parasite transmission in the community, community competence could increase. However, while viewing the microbiome as a species- or population-level trait can be useful for theoretical understanding, it oversimplifies the dynamics that actually occur within communities.

### Changes in microbiome composition at the individual level can alter community competence

In reality, traits related to microbiome composition (e.g., alpha and beta diversity, etc.) and changes therein vary among individuals within a species or population. Thus, changes in the microbiome composition of certain individuals (e.g., individual birds in Fig. 2C) can lead to either increased or decreased host quality of these individuals. A scenario like the one depicted in Fig. 2C could arise, for example, if environmental contamination disproportionately affects birds nesting closer to the contamination source, leading to changes in the microbiomes of only those individuals. The direction of the subsequent change in community competence would depend on the nature of the change in the microbiome. For instance, if microbiome composition shifts to include microbes that facilitate infection, there may be an increase in host quality (e.g., a shift toward “amplifier” or “super-spreader” status). Super-spreaders may increase community competence by serving as reservoirs for parasites or causing hosts to behave in ways that increase parasite transmission (Wanelik et al. 2024). Conversely, if microbiome composition changes to include microbes that protect against infection, there may be a decrease in host quality (e.g., a shift toward “dead-end host” or “non-host” status). If a large proportion of a species or population maintains a healthy microbiome, those hosts could act as a buffer against changes in parasite transmission dynamics, even if some individuals experience dysbiosis. In summary, the microbiome could be a pivotal, though overlooked, component underlying biodiversity–disease relationships. By taking the microbiome into account, we can begin to examine biodiversity–disease relationships in new and more comprehensive ways.

## Host-extrinsic factors impacting the microbiome may have knock-on effects

### Climate change

Climate change has been linked to changes in disease occurrence, and in some cases, the microbiome could act as a mediating factor. Both warming and cooling are generally associated with shifts in microbiome composition across taxa (Li et al. 2023) and can result either directly from temperature changes (Moeller et al. 2020) or indirectly, e.g., via temperature changes altering habitat availability (Watson et al. 2019). The microbiome has been shown to help provide thermal tolerance in non-model organisms including Pacific oysters (*Crassostrea gigas*) (Lokmer and Wegner 2015) and green frog (*Lithobates clamitans*) tadpoles (Fontaine et al. 2022). However, our overall understanding of how climate change affects the microbiome and parasite transmission remains limited (de Angeli Dutra et al. 2023). For example, while warming can increase the abundance of potentially parasitic microbes (Aydogan et al. 2018), the microbiome's ability to inhibit parasites may in some cases depend on the specific microbe strain and temperature in question (Muletz-Wolz et al. 2017), highlighting the need for more in-depth research. In addition to temperature changes, climate change-associated factors like rising sea levels (Kivistik et al. 2020) and changes in the frequency of severe weather events (Motes-Rodrigo et al. 2025) can impact host quality via the microbiome. Finally, while plant microbiomes will likely aid in short-term adaptation to climate change (Trivedi et al. 2022) and can generally help to mitigate abiotic stress (as discussed in the "Behavior and Abiotic Stress" section), it is also possible for climate change-related abiotic stressors (e.g., drought, flooding, and shifts in nutrient availability), to influence wild plant microbiomes in ways that potentially impact host quality (Hartman and Tringe 2019).

### Pollution

Anthropogenic pollution such as pesticides, microplastics, and heavy metals have been associated with shifts in microbiome composition across various taxa (e.g., Lapanje et al. 2010; Yu et al. 2023; Papazlatani et al. 2024), which may impact host quality. For instance, in the wild, contamination from sewage and urban runoff has been associated with higher infection rates in fish, which act as intermediate hosts for a nematode parasite transmissible to birds (Coyner et al. 2002). In this scenario, the microbiomes of either the fish or birds could play a role in parasite transmission by altering host quality. For example, if the fish have microbiomes that defend poorly against parasite colonization, or if bird microbiomes influence foraging behavior, there could be an increase in consumption of infected fish by birds. Contaminants also have the potential to directly introduce new diseases. For example, Southern sea otters (*Enhydra lutris nereis*) are exposed to *Toxoplasma gondii* likely through runoff carrying feline feces (Miller et al. 2002). The probability of infection once exposed, however, could hypothetically depend on sea otter microbiome composition. In some cases, the microbiome itself may serve as a buffer against the impacts of pollution, such as in insects that harbor microbes aiding in pesticide detoxification (Siddiqui et al. 2022).

### Land use change

Land use change can impact host microbiomes by altering both diet and *exposure* to environmental microbes, which can influence host quality. For instance, forest fragmentation has been associated with changes in microbiome composition in various species including non-human primates (e.g., Goldberg et al. 2008), potentially due to reduced or disrupted food availability. While changes in the microbiome can stem from variations in diet (e.g., Sugden et al. 2020), land use change can also alter the composition of soil microbiomes which can in turn affect both plant (Romdhane et al. 2022) and animal (Barnes et al. 2021) microbiomes via exposure to different environmental microbes. For example, habitat fragmentation can lead to changes in soil microbiomes that impact plant performance and investment (Kiesewetter and Afkhami 2021), which may then influence host quality. Other types of land use change, like crop rotation, have been shown to suppress disease by affecting microbiome composition (Peralta et al. 2018), illustrating how land use change may in some cases decrease rather than increase host quality. In addition, a number of infectious diseases have emerged by spillover of parasites from domestic to wild animals (Daszak et al. 2001), which could be in part facilitated by microbiome differences seen in hosts along urbanization gradients (e.g., Mistrick et al. 2024).

### Captivity and domestication

Captivity can alter host microbiomes through mechanisms such as altered diet, exposure to a more homogenous environment, altered intra- or inter-specific interactions, increased stress, and antibiotic use. Captive animals are particularly susceptible to disease, including gastrointestinal and metabolic disorders, which may be mediated via changes to the microbiome (Williams et al. 2019). For example, captive boreal toads (*Anaxyrus boreas*) exhibited lower microbiome diversity and increased susceptibility to *Batrachochytrium dendrobatidis*, the fungus causing chytridiomycosis, in comparison to wild toads (Kueneman et al. 2016). However, while captivity is often associated with lower microbiome diversity (Williams et al. 2019), the microbiomes of captive animals differ from those of their wild counterparts in non-systematic ways (McKenzie et al. 2017; Alberdi et al. 2021). Thus, it is impossible to generalize the effects of captivity on host quality via the microbiome, highlighting the need for additional research.

In many cases, plant domestication has not been shown to impact microbiome diversity but rather to alter microbiome composition and abundance, including that of functional microbial taxa (Gutierrez and Grillo 2022; Coleman-Derr et al. 2016). For example, multiple studies have found a reduction in symbiotic mycorrhizal fungi and nitrogen-fixing bacteria in the root microbiome of domesticated plants (Pérez-Jaramillo et al. 2018). Shi et al. (2019) provided a more direct link to host quality by showing that wild crops can have a higher abundance of beneficial symbionts and a lower abundance of parasites compared to their cultivated varieties, suggesting that domestication-related differences in microbiomes may play a mediating role in host quality.

### Future research directions

Advancing knowledge of how host–microbiome interactions influence biodiversity–disease relationships in animal and plant communities will require integrating empirical evidence, theoretical frameworks, and analytical tools. While studying these interactions at the community level is challenging, key components, as identified here, can be examined individually: (1) the microbiome's impact on host quality (Fig. 1A), (2) how such impacts affect parasite transmission (Fig. 1B), and (3) the effects of anthropogenic change on the microbiome (Fig. 1C). To address the first point, expanding microbiome research across diverse plant and animal species in both healthy and diseased states is essential. Current data are largely correlative, making causal inferences difficult. Moving beyond descriptive studies to approaches aimed at understanding mechanisms, such as experimental (e.g., microbiome manipulations and germ-free models) and theoretical (e.g., mathematical modeling) approaches, will help establish causal links between microbiome composition and host quality. We noted that certain host-associated factors seem to correlate more strongly with specific aspects of host quality (e.g., reproduction and behavior are primarily linked to *exposure*), and further research will help reveal whether these relationships hold or if new patterns emerge. Additionally, incorporating microbiomes into ecological and epidemiological models, similar to how prior studies have integrated components like social interaction into **SIR models** (Martignoni et al. 2024, see Table 1, Glossary), may provide novel insights into disease dynamics.

To better understand the impacts of microbiomes on biodiversity–disease relationships, experiments at multiple scales are needed. Studies at ecologically relevant time scales (e.g., across annual cycles, seasons, or years) and at different levels of biological organization (e.g., at individual and ecosystem levels) would be valuable. Longitudinal studies could reveal how seasonal microbiome changes affect host quality and disease outcomes or how microbiomes influence infection progression and recovery. Integrating multiple biological scales could help unravel complexities in disease ecology, as interactions above and below the population level are interconnected but poorly understood (Tompkins et al. 2011; Johnson et al. 2015). Additionally, studying both animal and plant microbiomes together could deepen our understanding of parasite transmission, as plants and animals share microbial reservoirs, environmental influences, and, in some cases, parasite transmission pathways.

Our illustration of how anthropogenic change can influence biodiversity–disease relationships via microbiome shifts is overly simplistic due to a dearth of existing data. It is established that some results of anthropogenic global change, including biodiversity loss, are associated with increased disease risk (Mahon et al. 2024). However, microbiome changes could either enhance or impair host quality, thus creating additive (i.e., working together with biodiversity loss to increase disease risk) or opposing (i.e., working in opposition to biodiversity loss to reduce disease risk) effects with changes in community composition. Ultimately, further research is needed to elucidate the effects of different types of anthropogenic change on microbiomes, as well as potential cascading impacts to host quality and parasite transmission. Additionally, microbiome data could help better inform disease management strategies and thus mitigate negative effects of anthropogenic change. For example, while buffer zones established around diseased populations may help prevent parasite transmission between species, they may also limit microbiome exposure, potentially hindering the development of diverse and healthy microbiomes. Overall, experimental studies across diverse wild species are needed to clarify such dynamics.

## Conclusion

Understanding the intricate relationships between the microbiome, host quality, and parasite transmission is essential for a deeper comprehension of ecological interactions. Moving forward, a more holistic approach that considers within-individual biodiversity, specifically the microbiome, can challenge and refine our understanding of biodiversity–disease relationships and disease ecology as a whole. By integrating microbiome dynamics into disease ecology frameworks, we may uncover novel insights into how microbial communities shape host health and disease outcomes.

## Data Availability

No new data were generated or analyzed in this article.

## Notes

From the symposium “Paddling Together: Navigating the Crosscurrents of Plant and Animal Biology to Explore Uncharted Waters in Disease Ecology” presented at the annual meeting of the Society for Integrative and Comparative Biology, January 3–7, 2025 at Atlanta, Georgia.
